# Fown’s Manual of Chemistry

**Published:** 1886-01

**Authors:** 


FOWNS' MANUAL OF CHEMISTRY. Theoretical and practical. A New American from the Twelfth English Edition, embodying Watts' "Physical and Inorganic Chemistry," with one hundred and sixty-eight illustrations. Philadelphia: Lea Brothers & Co. 1885.

This work needs no extended review at our hands. The fact that it has reached its twelfth edition is the best proof of its value as a text-book.

